# Lipoteichoic Acid Fraction from *Lactiplantibacillus plantarum* K8 Attenuates Inflammatory Responses and Promotes Antimicrobial Defense in Oral Epithelial Cells

**DOI:** 10.3390/microorganisms14061255

**Published:** 2026-06-02

**Authors:** Inseong Hwang, Gyubin Jung, Hangeun Kim, Dae-Kyun Chung

**Affiliations:** 1 Graduate School of Biotechnology, Kyung Hee University, Yongin 17104, Republic of Korea; inshwang@khu.ac.kr (I.H.); herbdoctor97@khu.ac.kr (G.J.); 2 Research and Development Center, Skin Biotechnology Center Co., Ltd., Yongin 17104, Republic of Korea; 3 Skin Biotechnology Center, Kyung Hee University, Suwon 16229, Republic of Korea

**Keywords:** periodontitis, postbiotics, lipoteichoic acid, anti-inflammation, TLR2, IRAK-M, antimicrobial peptide

## Abstract

Gingivitis, periodontitis, and stomatitis are common oral inflammatory disease affecting a large proportion of the global population. Increasing attention has recently been given to the development of health functional materials aimed at maintaining oral health and preventing microbial-associated oral disease. This study evaluated the efficacy of the lipoteichoic acid (LTA) fraction derived from the probiotic *Lactiplantibacillus plantarum* K8 (pLF) in preventing oral inflammation and microbial infection using the oral epithelial cell line YD-38. The results confirmed that pLF enhances the expression of interleukin-1 receptor-associated kinase M (IRAK-M), a negative regulator of Toll-like receptor (TLR) signaling, and inhibits the expression of pro-inflammatory cytokines, including C-C motif ligand 2 (CCL2), interleukin-6 (IL-6), and interleukin-8 (IL-8), in YD-38 cells stimulated with tumor necrosis factor-α (TNF-α) and interferon-γ (IFN-γ). Furthermore, it was demonstrated that pLF induces IRAK-M expression in a TLR2-involved manner and inhibits nuclear factor-kappa B (NF-κB) signaling, thereby reducing the expression of pro-inflammatory cytokines. pLF also exhibits oral antimicrobial efficacy by increasing the expression of the antimicrobial peptide human β-defensin 1 (hBD1) and human β-defensin 2 (hBD2) in a TLR2-involved manner and effectively inhibiting the growth of *Porphyromonas gingivalis* and *Staphylococcus aureus* in the epithelial cell associated system. Therefore, the LTA fraction derived from *L. plantarum* K8 represents a promising postbiotic candidate for the regulation of oral immune and microbial responses.

## 1. Introduction

Oral inflammatory diseases, including gingivitis, periodontitis, and stomatitis, are highly prevalent conditions that contribute substantially to oral disease burden worldwide [1]. They remain a major cause of chronic oral inflammatory disease and have been increasingly recognized as being associated with systemic conditions such as cardiovascular disease, diabetes, and adverse pregnancy outcomes. These associations suggest that oral inflammatory status may contribute to systemic health through a chronic inflammatory burden and the dissemination of microbial and inflammatory mediators from the oral cavity [2]. The oral epithelium plays a critical role in this context, serving not merely as a passive physical barrier but as an active immunological interface. It is continuously exposed to a diverse and dense microbial consortium, requiring a sophisticated regulatory mechanism to distinguish between symbiotic commensals and opportunistic pathogens. This dynamic balance is maintained through tightly regulated innate immune signaling networks that allow epithelial cells to rapidly respond to microbial challenges while preventing excessive inflammatory activation.

Gingivitis is a reversible inflammatory condition of the gingival tissue primarily induced by dental plaque accumulation. It represents an early stage of gingival inflammation characterized by localized immune activation that remains confined to soft periodontal tissues. When oral hygiene is not adequately maintained, persistent inflammatory stimulation can sustain host immune responses and increase susceptibility to disease progression. If left untreated, gingivitis may progress to periodontitis, a chronic inflammatory disease characterized by irreversible destruction of periodontal supporting structures, including the periodontal ligament and alveolar bone, ultimately leading to tooth loss [3].

Periodontitis is primarily driven by dysbiotic microbial communities that form structured biofilms on tooth surfaces, promoting pathogenic colonization and excessive host immune responses, which result in persistent inflammation and tissue destruction. This pathogenic shift often triggers an overproduction of reactive oxygen species (ROS) and Matrix Metalloproteinases (MMPs) within the periodontal pockets, which contribute to degradation of the extracellular matrix and may exacerbate bone resorption pathways. Sustained host–microbe interactions within this biofilm environment further amplify inflammatory signaling and contribute to progressive periodontal tissue damage [4]. These processes are further reinforced by continuous recruitment of immune cells and sustained cytokine release, which collectively contribute to chronic inflammatory amplification within periodontal tissues.

Similarly, stomatitis refers to inflammatory conditions of the oral mucosa arising from infectious, autoimmune, allergic, or nutritional factors. Despite its heterogeneous etiology, stomatitis is commonly characterized by disruption of epithelial integrity and dysregulated inflammatory responses within the oral mucosal environment. These alterations can impair mucosal barrier function and delay tissue repair processes, thereby prolonging inflammatory conditions [5,6]. The recurrent nature of oral mucosal lesions often inflicts profound discomfort and psychological stress on patients, reducing their quality of life. Current clinical interventions primarily rely on topical corticosteroids or broad-spectrum antiseptics; however, their long-term utilization is frequently limited by adverse secondary effects, including mucosal atrophy and the disruption of the natural oral microflora balance. Consequently, there is an urgent unmet medical need for non- steroidal, biocompatible therapeutic alternatives.

A key factor in the development of oral inflammatory disease is dental plaque biofilm, which consists of complex microbial communities that facilitate pathogen persistence and sustain inflammatory responses. Therefore, effective strategies targeting both microbial balance and host epithelial responses are essential for maintaining oral health.In recent years, probiotics have gained increasing attention as potential modulators of oral microbial ecology and host-microbe interactions [7,8]. Probiotic-derived components can influence microbial composition, inhibit pathogen colonization, and suppress biofilm formation. These effects are mediated through multiple mechanisms, including competition with pathogenic species, modulation of microbial adhesion, and interference with biofilm maturation processes within the oral environment. In particular, lipoteichoic acid derived from *Lactiplantibacillus plantarum* has been reported to inhibit multispecies oral pathogenic biofilm formation [9,10] suggesting a potential role in disrupting pathogenic biofilm development at an early stage. Moreover, several probiotic strains, including *Lactiplantibacillus* and *Bifidobacterium* species, produce antimicrobial molecules such as bacteriocins that suppress periodontal pathogens, including *Porphyromonas gingivalis* and *Aggregatibacter actinomycetemcomitans* [11,12]. These probiotic associated factors may contribute not only to direct antimicrobial activity but also to the modulation of local microbial communities toward a less pathogenic state.

However, despite these promising findings, the application of live probiotics presents several limitations, including concerns regarding safety, stability, and regulatory constraints. In particular, live microorganisms may exhibit variable viability during manufacturing, storage, and gastrointestinal transit, which can compromise their reproducibility and therapeutic efficacy. In addition, potential risks such as opportunistic infection in immunocompromised individuals and difficulties in standardizing dosage further limit their clinical applicability. Furthermore, transferring live bacteria into an already inflamed, highly vascularized periodontal tissue poses a theoretical risk of bacteremia, which demands extreme caution. 

To overcome these limitations, postbiotics–defined as non-viable microbial cells, cell components, or metabolites that confer health benefits–have emerged as attractive alternatives [13,14]. Previous studies have suggested the potential involvement of postbiotic-derived components in host immune modulation and microbial regulation [15,16]. Crucially, postbiotics exhibit superior chemical stability, a longer shelf-life, and precise dosage control, making them exceptionally suited for topical oral care formulations such as mouthwashes, dentifrices, and bio adhesive gels. However, their functional contributions to epithelial immune regulation and antimicrobial defense in the oral environment remain poorly understood. In particular, the mechanisms by which postbiotic-derived factors modulate epithelial inflammatory and antimicrobial responses have not been clearly defined. This includes responses associated with epithelial innate immune sensing and downstream inflammatory signaling events in the oral mucosal environment. While pattern recognition receptor-mediated pathways are likely to be involved in host recognition of microbial-derived components [17,18], the specific signaling mechanisms underlying these effects in oral epithelial cells remain largely unclear.

Therefore, this study aimed to investigate whether a purified lipoteichoic acid fraction derived from *Lactiplantibacillus plantarum* K8 (pLF) can regulate inflammatory responses and enhance antimicrobial defense in human gingival epithelial cells (YD-38). Specifically, we examined the involvement of receptor-mediated signaling in epithelial immune regulation, including its effects on negative regulators of inflammatory signaling, NF-κB activation, pro-inflammatory cytokine production, and antimicrobial peptide gene expression, thereby evaluating the potential of pLF as a functional postbiotic candidate for the regulation of oral inflammatory and antimicrobial responses.

## 2. Materials and Methods

### 2.1. YD-38 Cell Culture and Experimental Conditions

Human oral squamous cell carcinoma-derived epithelial cells, YD-38, were maintained in RPMI 1640 medium (Welgene, Daegu, Republic of Korea) supplemented with 10% fetal bovine serum (FBS; Gibco, Thermo Fisher Scientific, Waltham, MA, USA) and 1% penicillin–streptomycin (P/S; GenDEPOT, Katy, TX, USA) at 37 °C in a humidified atmosphere containing 5% CO_2_. Cells were subcultured every 2−3 days using trypsin-EDTA (Welgene) and seeded onto appropriate culture plates (6-well or 96-well plates) and incubated overnight to allow for complete cell attachment and stabilization before any experimental treatment. To induce inflammatory responses, cells were stimulated with recombinant human TNF-α and IFN-γ (each 10 ng/mL; R&D systems, Minneapolis, MN, USA) referred to as TI. To inhibit Toll-like receptor 2 (TLR2)-mediated signaling, cells were pretreated with a neutralizing anti-human TLR2 monoclonal antibody (Anti-hTLR2-IgA2; In vivo Gen, San Diego, CA, USA) at 5 µg/mL for 1 h prior to treatment with the K8 LTA fraction.

### 2.2. Preparation of K8 LTA Fraction (pLF)

The lipoteichoic acid fraction derived from *L. plantarum* K8 (pLF) was prepared as previously described [19]. *L. plantarum* K8 (KCTC 10887BP) was cultured in MRS broth at 37 °C for 16 h. Cells were harvested by centrifugation at 4000× *g* for 10 min and resuspended in 0.1 M sodium citrate buffer (pH 4.7). Bacteria cells were disrupted by ultrasonication, followed by extraction with an equal volume of *n*-butanol with stirring for 30 min at room temperature. After centrifugation at 13,000× *g* for 20 min, the aqueous phase was collected, dialyzed against pyrogen free water, and further purified using hydrophobic interaction chromatography on an octyl-Sepharose CL-4B column (2.5 × 20 cm, Sigma Aldrich, St. Louis, MO, USA). Elution was performed using a stepwise n-propanol gradient (20%, 35%, and 45%). Fractions containing LTA were pooled based on inorganic phosphate assay, dialyzed against distilled water, and designated as LTA fraction (pLF). 

### 2.3. Cell Viability Assay

YD-38 cells were seeded in 96-well plates at 1 × 10^5^ cells/mL and incubated overnight. Cells were then treated with increasing concentrations of pLF up to 100 µg/mL in serum-free medium for 24 h. After treatment, MTT solution (500 µg/mL; Invitrogen, Waltham, MA, USA, Thermo Fisher Scientific) was added and incubated for 30 min to allow viable cells to metabolize the substrate into insoluble formazan. Formazan crystals were dissolved in 100 µL DMSO, and absorbance was measured at 550 nm using an Epoch Microplate Spectrophotometer (BioTek Instruments, Winooski, VT, USA).

### 2.4. RNA Extraction and RT-qPCR Analysis 

YD-38 cells were seeded in 6-well plates and treated with pLF (up to 100 µg/mL) for 18 h with or without TI stimulation. For time-course analysis, cells were treated with pLF for 0, 3, 6, 12, and 24 h. Total RNA was extracted using the easy- BLUE™ Total RNA Extraction Kit (iNtRON Biotechnology, Seongnam, Republic of Korea), and cDNA was synthesized using PrimeScript™ RT Master MIX (TaKaRa Bio INC., Shiga, Japan). RT-qPCR was performed using TB Green® Premix Ex Taq™ II (TaKaRa) on an AriaMx Real-Time PCR System (Agilent Technologies, Santa Clara, CA, USA). Gene expression levels were normalized to human GAPDH (hGAPDH). Primer sequences are listed in Table S1. For each experimental condition, samples were processed in parallel to minimize batch-to-batch variation, and identical reagent lots were used within the same experimental set whenever possible.

### 2.5. Enzyme-Linked Immunosorbent Assay

YD-38 cells were treated with pLF (100 µg/mL) for 18 h and subsequently stimulated with TI for 24 h. Culture supernatants were collected and analyzed for IL-6 and IL-8 production using BD OptEIA Human ELISA kits (BD Biosciences, San Jose, CA, USA) according to the manufacturer’s instructions. For quantification, Samples were diluted 1:2 for IL-6 and 1:20 for IL-8 prior to analysis.

### 2.6. Western Blot Analysis

YD-38 cells were treated with pLF (100 µg/mL) for 18 h followed by TI stimulation for 0, 15, 30, 60, and 120 min. Cells were lysed using 2× Laemmli buffer and proteins were separated by SDS-PAGE (12%). Proteins were transferred to PVDF membranes (Cytiva, Marlborough, MA, USA) and blocked with 5% skim milk in TBST for 1 h at room temperature. To ensure a through removal of unbound blocking reagents, the membranes were rinsed three times with fresh TBST buffer before primary antibody incubation. Membranes were incubated overnight at 4 °C with primary antibodies against phospho-ERK1/2, phospho-p38, phospho-p65, and phospho-STAT1 (Cell Signaling Technology, Danvers, MA, USA). After washing, HRP-conjugated secondary antibody (Santa Cruz Biotechnology, Dallas, TX, USA) was applied for 2 h at room temperature. Protein bands were visualized using EZ-Western Lumi Pico (DoGenBio, Seoul, Republic of Korea) and quantified using ImageJ software (Version 1.54g, National Institutes of Health, Bethesda, MD, USA). β-actin was used as a loading control.

### 2.7. Staphylococcus aureus Infection Assay

YD-38 cells were seeded in antibiotic-free RPMI medium and pretreated with pLF (100 µg/mL) for 24 h at 37 °C in a humidified 5% CO2 incubator. *Staphylococcus aureus* (ATCC 29523) was cultured in BHI broth and added to cells at 1 × 10^8^ CFU/mL for 6 h infection. After infection, extracellular bacteria were removed using gentamicin (100 µg/mL), and cells were lysed using 0.1% Triton X-100. Lysates were plated on BHI agar plates and incubated at 37 °C overnight for colony counting.

### 2.8. Bactericidal Activity of Conditioned Media Against Porphyromonas gingivalis

Conditioned media (CM) were collected from YD-38 cells treated with pLF (100 µg/mL) for 48 h. *Porphyromonas gingivalis* (KCTC 5352) was cultured anaerobically in BHI broth at 37 °C for 72 h. CM (900 µL) was mixed with 100 µL bacterial suspension (1 × 10^9^ CFU/mL) and incubated for 6 h under anaerobic conditions in an anaerobic chamber. The mixture was plated on BHI agar and incubated anaerobically at 37 °C for 3 days. Colony-forming units (CFU) were counted. Gentamicin (50 µg/mL) served as positive control.

### 2.9. Statistical Analysis

All experiments measurements were conducted in technical triplicates. Data are expressed as mean ± standard deviation (SD). Statistical significance was analyzed using Student’s *t*-test or one-way ANOVA followed by Tukey’s post hoc test. A *p*-value < 0.05 was considered statistically significant. Graphs were generated using GraphPad Prism 8 (GraphPad Software, San Diego, CA, USA).

## 3. Results

### 3.1. pLF Exhibits No Cytotoxicity in YD-38 Cells

**Figure 1:**
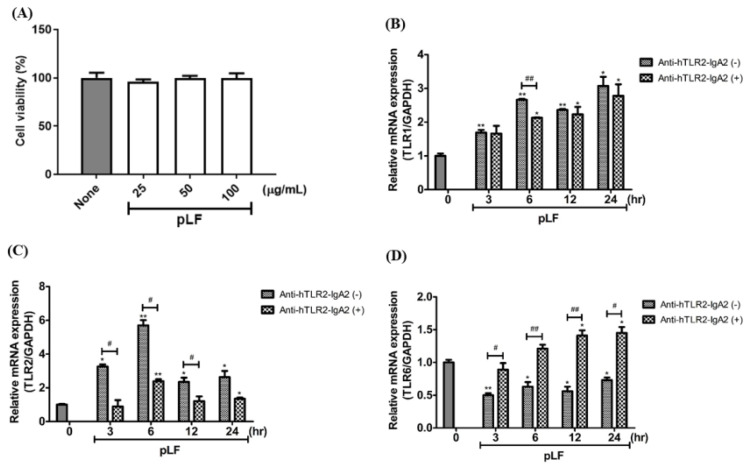
Cell viability and receptor-related gene expression in YD-38 cells. (**A**) Cell viability of YD-38 cells treated with pLF (25, 50, and 100 μg/mL) for 24 h, determined by MTT assay. mRNA expression of (**B**) TLR1, (**C**) TLR2, and (**D**) TLR6 in YD-38 cells treated with pLF (100 μg/mL) in the presence or absence of a TLR2 neutralizing antibody. Data are presented as mean ± SD (*n* = 3). * *p* < 0.05, ** *p* < 0.01 compared to none, # *p* < 0.05, ## *p* < 0.01 compared to anti-hTLR2-igA2 (–).

YD-38 cells were treated with pLF at concentrations of 25, 50, and 100 μg/mL for 24 h to evaluate cytotoxicity. As shown in Figure 1A, pLF treatment did not significantly affect cell viability at any tested concentration, with all groups maintaining viability levels comparable to the untreated control. These results indicate that pLF is non-cytotoxic to YD-38 cells under the experimental conditions, and concentrations up to 100 μg/mL were used for subsequent experiments. The absence of cytotoxicity was further supported by microscopic observation, which showed no apparent morphological alterations in treated cells across all conditions.

### 3.2. pLF Differently Regulates TLR1/2/6 Expression Involved with TLR2 Signaling 

To investigate whether pLF modulates Toll-like receptor (TLR) expression, the mRNA levels of TLR1, TLR2, and TLR6 were analyzed in YD-38 cells following pLF treatment. pLF treatment resulted in a time-dependent increase in TLR1 (Figure 1B) and TLR2 (Figure 1C) expression, whereas TLR6 expression was reduced (Figure 1D). The increase in TLR2 expression was most pronounced at 6–24 h, suggesting a cell-surface receptor reprogramming event. To determine whether these effects were mediated by TLR2 signaling, cells were pretreated with a TLR2 neutralizing antibody. The antibody effectively suppressed the pLF-induced upregulation of TLR2 (Figure 1C). In addition, the reduction in TLR6 expression observed after pLF treatment was reversed in the presence of the neutralizing antibody (Figure 1D). These findings suggest that TLR2 is involved in the regulation of TLR expression induced by pLF. Overall, these results indicate that pLF modulates the expression pattern of TLR family members in a time-dependent manner in YD-38 cells.

### 3.3. pLF Induces IRAK-M Expression with the Involvement of TLR2 Signaling 

**Figure 2:**
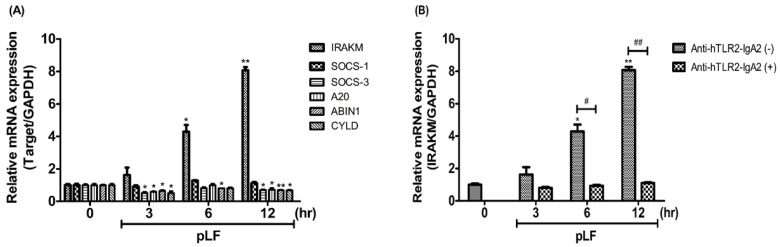
Expression of negative regulators in YD-38 cells. (**A**) mRNA expression of negative regulators in YD-38 cells treated with pLF 100 μg/mL. (**B**) mRNA expression of IRAK-M in YD-38 cells pretreated with a TLR2 neutralizing antibody prior to pLF 100 μg/mL treatment. * *p* < 0.05, ** *p* < 0.01 compared to none, # *p* < 0.05, ## *p* < 0.01 compared to anti-hTLR2-igA2 (–).

To identify negative regulators involved in TLR signaling, the mRNA expression levels of several regulatory molecules were examined following pLF treatment (Figure 2A). Among the tested genes, IRAK-M expression was increased in a time-dependent manner, showing a 4.3-fold increase at 6 h and reaching an 8.1-fold increase at 12 h compared with the untreated control. In contrast, other negative regulators, including SOCS-1, SOCS-3, A20, ABIN1, and CYLD exhibited no significant changes throughout the entire testing period. Furthermore, pretreatment with a TLR2 neutralizing antibody significantly attenuated the induction of IRAK-M expression with 8.1-fold increase at 12 h (Figure 2B). These results suggest that TLR2 signaling is involved in pLF-induced IRAK-M expression. 

### 3.4. pLF Suppresses NF-κB Signaling Pathways

**Figure 3:**
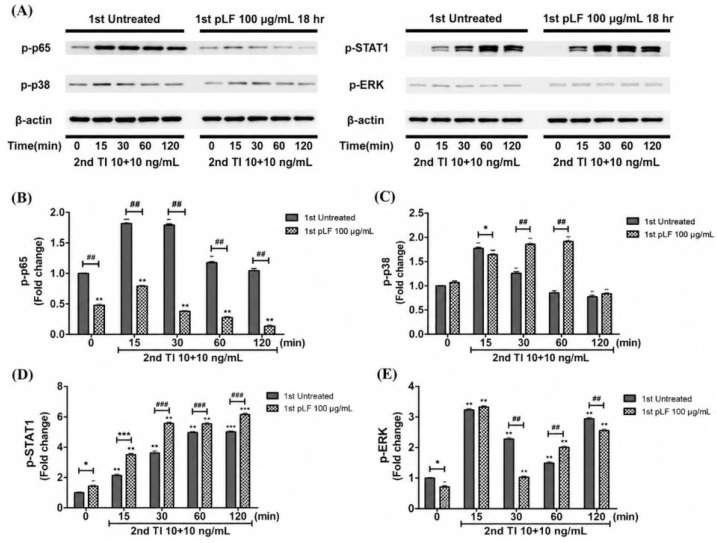
Analysis of signaling protein phosphorylation. (**A**) Representative Western blot images of phosphorylated signaling proteins in YD-38 cells pretreated with pLF 100 μg/mL followed by TI stimulation. (**B**) Quantification of phosphorylated p65 levels (**C**) Quantification of phosphorylated p38 levels. (**D**) Quantification of phosphorylated STAT1 levels (**E**) Quantification of phosphorylated ERK levels normalized to β-actin. * *p* < 0.05, ** *p* < 0.01, *** *p* < 0.001, compared to none, ## *p* < 0.01, ### *p* < 0.001 compared to untreated.

To further investigate the downstream signaling pathways, the phosphorylation levels of NF-κB p65, p38 MAPK, STAT1, ERK proteins were analyzed by Western blot (Figure 3A). pLF pretreatment significantly reduced the phosphorylation of NF-κB p65 at all examined time points compared to untreated controls (Figure 3B), indicating effective suppression of NF-κB activation. In contrast, p38 MAPK phosphorylation was transiently increased at early time points (Figure 3C). Phosphorylation of STAT1 was markedly increased in response to TI stimulation and was further enhanced in pLF treatment compared to untreated controls (Figure 3D). ERK phosphorylation exhibited a time-dependent pattern, showing reduced levels at 30 min, a transient increase at 60 min, and a decrease again at 120 min in pLF treatment compared to untreated controls (Figure 3E). These results suggest that pLF suppresses NF-κB signaling while differentially modulating MAPK and STAT1 pathways.

### 3.5. pLF Inhibits Pro-Inflammatory Cytokine Expression

**Figure 4:**
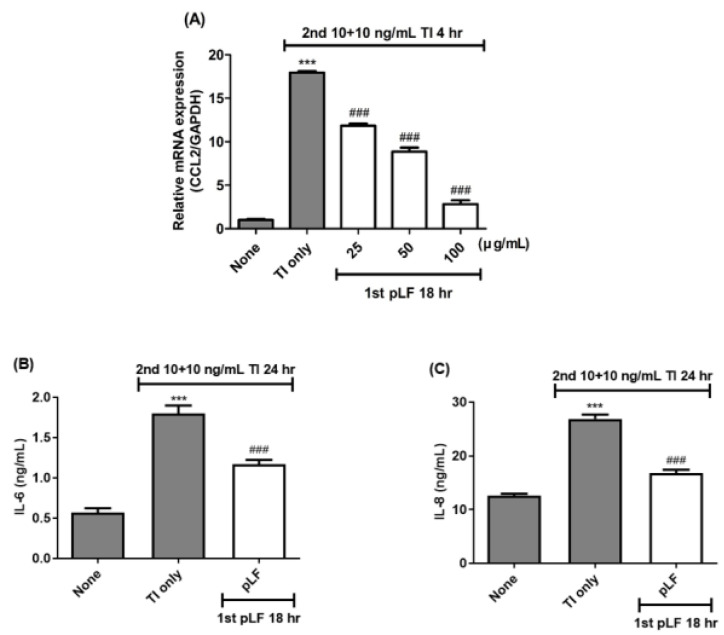
Pro-inflammatory cytokine expression in YD-38 cells. (**A**) mRNA expression of CCL2 in TI-stimulated YD-38 cells with or without pLF 100 μg/mL pretreatment. (**B**) Protein levels of IL-6 measured by ELISA. (**C**) Protein levels of IL-8 measured by ELISA. *** *p* < 0.001 compared to none, ### *p* < 0.001 compared to TI only.

The anti-inflammatory effect of pLF was further evaluated by measuring the expression of pro-inflammatory cytokines in TI-stimulated YD-38 cells. TI stimulation significantly increased the mRNA expression of CCL2 up to 17.8-fold change compared with the untreated control (Figure 4A). However, pretreatment with pLF reduced CCL2 expression in a concentration-dependent manner, with the highest concentration (100 μg/mL) decreasing expression to 2.7-fold change. Consistent with these findings, ELISA results showed that the protein levels of IL-6 (Figure 4B) and IL-8 (Figure 4C) were significantly elevated following TI stimulation, whereas pLF pretreatment markedly reduced their expression. These findings demonstrate that pLF effectively suppresses pro-inflammatory cytokine production.

### 3.6. pLF Enhances Antimicrobial Peptide mRNA Expression with the Involvement of TLR2 Signaling

**Figure 5:**
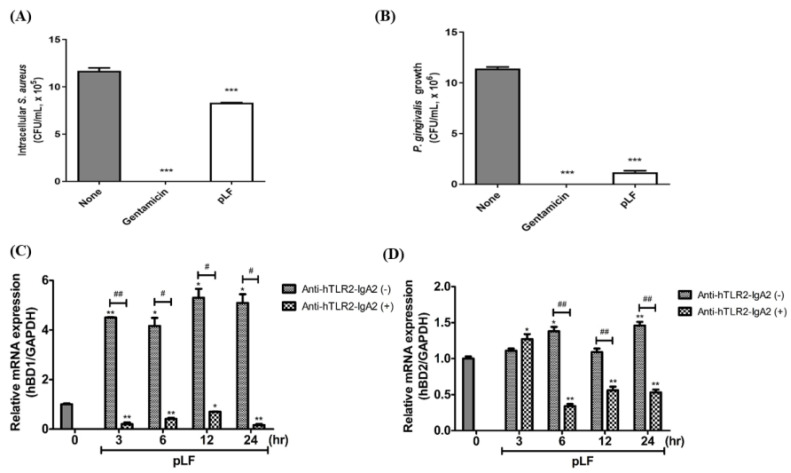
Antimicrobial responses induced by pLF 100 μg/mL in YD-38 cells. (**A**) Intracellular *Staphylococcus aureus* CFU following pLF 100 μg/mL pretreatment and infection with 1 × 10^8^CFU/mL. (**B**) Growth of *Porphyromonas gingivalis* in conditioned media derived from 100 μg/mL pLF-treated cells. mRNA expression of (**C**) hBD1 and (**D**) hBD2 with or without TLR2 neutralization. * *p* < 0.05, ** *p* < 0.01, *** *p* < 0.001 compared to none, # *p* < 0.05, ## *p* < 0.01 compared to anti-hTLR2-igA2 (–).

To assess the antimicrobial response, the mRNA expression levels of human β-defensins was analyzed in YD-38 cells. pLF treatment increased hBD1 mRNA expression in a time-dependent manner (Figure 5C). An increase in hBD2 mRNA expression was also observed (Figure 5D). However, pretreatment with a TLR2 neutralizing antibody reduced the mRNA expression levels of both hBD1 (Figure 5C) and hBD2 (Figure 5D). These results suggest that TLR2 signaling is involved in pLF-induced antimicrobial peptide mRNA expression.

### 3.7. pLF Exhibits Antibacterial Activity Against Oral Pathogens 

To evaluate the antibacterial activity of pLF, intracellular infection and bacterial growth inhibition assays were performed. In YD-38 cells infected with *S. aureus*, intracellular bacterial counts were increased in the untreated infected group. In contrast, pLF pretreatment reduced intracellular CFU levels. In addition, conditioned media collected from pLF-treated YD-38 cells inhibited the growth of *P. gingivalis* compared with bacteria cultured in fresh media (untreated control). The inhibitory effect of pLF-derived conditioned media was also comparable to that of gentamicin treatment. These results indicate that pLF treatment is associated with enhanced antibacterial effects in both intracellular *S. aureus* infection and extracellular *P. gingivalis* growth conditions. 

## 4. Discussion

This study demonstrates that the lipoteichoic acid fraction derived from *L. plantarum* K8 (pLF) exerts both anti-inflammatory and antimicrobial effects in human oral epithelial cells, with involvement of TLR2-related signaling. These findings are consistent with previous reports showing that *L. plantarum* K8 lysates exhibit anti-inflammatory activity in the oral environment [20,21], and further support the growing evidence that postbiotic components can retain biological functionality while overcoming the limitations associated with live probiotics [22,23]. By utilizing a purified cell wall component rather than whole-cell lysates, this study provides more targeted molecular insight, minimizing confounding biological complexity and highlighting the potential therapeutic relevance of cell-surface amphiphilic components, particularly lipoteichoic acid. Such an approach also allows better attribution of observed immune responses to specific microbial molecular structures, which is important for understanding host-microbe interaction specificity.

A key finding of this study is that pLF induces the expression of IRAK-M, a well-known negative regulator of TLR signaling, suggesting that IRAK-M may be associated with downstream modulation of NF-κB activity, although the precise mechanistic hierarchy within epithelial signaling networks remains to be fully elucidated.

Unlike other IRAK family members that propagate downstream cascades, IRAK-M suppresses signaling by interfering with IRAK complex formation, thereby attenuating NF-κB activation [24,25]. In agreement with this mechanism, pLF treatment resulted in reduced phosphorylation of NF-κB p65, indicating suppression of pro-inflammatory signaling. These findings suggest that pLF contributes to the regulation of inflammatory responses and may support the maintenance of immune homeostasis rather than acting as a direct inhibitor of inflammation. The time-dependent induction of IRAK-M mRNA, reaching up to 8.1-fold at 12 h, corresponded with reduced NF-κB activation observed in Western blot analysis, suggesting an association between IRAK-M expression and modulation of inflammatory signaling in oral epithelial cells. This regulatory pattern may represent a feedback mechanism by which epithelial cells limit excessive inflammatory responses while maintaining responsiveness to microbial-derived stimuli.

Furthermore, pLF also influenced additional signaling pathways, as evidenced by increased STAT1 phosphorylation and modulation of MAPK signaling, while NF-κB suppression remained the predominant response. The increase in STAT1 phosphorylation, which is generally associated with interferon-mediated antimicrobial and antiviral responses, suggests that pLF may contribute to broader modulation of epithelial immune signaling. This pattern, characterized by attenuation of NF-κB activity while maintaining STAT1-associated signaling, indicates that pLF functions as an immunomodulatory factor rather than a general immunosuppressive agent. In addition, pLF modulated TLR expression by increasing TLR1 and TLR2 while decreasing TLR6 expression, and these effects were reduced following TLR2 neutralization. Although the functional implications of this differential regulation require further investigation, these findings suggest that pLF may influence TLR dimer expression balance and downstream signaling specificity. While TLR2 involvement was supported by neutralization experiments, additional studies are required to clarify receptor-level mechanisms.

Importantly, pLF significantly suppressed the expression of pro-inflammatory cytokines, including CCL2, IL-6, and IL-8, in response to inflammatory stimulation. These cytokines are known to contribute to immune cell recruitment and amplification of inflammatory responses in periodontal disease. For example, IL-8 is a major chemoattractant for neutrophils, and excessive neutrophil infiltration is associated with tissue damage in periodontitis. Similarly, CCL2 is involved in macrophage recruitment and contributes to chronic inflammatory progression. Quantitatively, pLF reduced the TI-induced increase in CCL2 from 17.8-fold to 2.7-fold, indicating a substantial attenuation of inflammatory signaling at the epithelial level. Overall, these results suggest that pLF modulates epithelial-derived inflammatory mediators involved in leukocyte recruitment.

In parallel with its anti-inflammatory effects, pLF enhanced the mRNA expression of antimicrobial peptides, particularly human β-defensin 1 and 2. These peptides are key components of epithelial innate immune responses and are involved in host defense against oral pathogens such as *P. gingivalis* [26]. Importantly, the simultaneous suppression of pro-inflammatory cytokines and enhancement of antimicrobial peptide mRNA expression suggests that pLF modulates epithelial immune responses without attenuating the transcriptional activation of innate defense-related genes. This dual functional profile may contribute to maintaining a balanced epithelial response, addressing a limitation of conventional anti-inflammatory agents that can broadly suppress immune signaling and potentially impair local antimicrobial defense mechanisms.

Furthermore, pLF-treated epithelial cells showed increased antibacterial activity against both Gram-positive and Gram-negative bacteria, including *S. aureus* and *P. gingivalis*. The oral cavity has been reported to serve as a reservoir for *S. aureus*, including antibiotic-resistant strains [27,28], highlighting the potential relevance of these findings. In addition, conditioned media from pLF-treated cells inhibited the growth of *P. gingivalis*, suggesting that soluble factors induced by pLF contribute to antimicrobial effects. Given that hBD1 and hBD2 are secreted antimicrobial peptides, these results are consistent with transcriptional activation of innate defense molecules; however, direct protein-level quantification was not performed.

Notably, the coordinated regulation of inflammatory and antimicrobial responses observed in this study suggests that pLF may act as a functional microbial-derived modulator that fine-tunes epithelial innate immune activity rather than simply suppressing inflammation. This integrated response pattern is consistent with receptor-mediated signaling dynamics, in which TLR2-associated pathways contribute to both inflammatory attenuation and antimicrobial gene activation. Such a mechanism may be particularly relevant in the oral environment, where epithelial cells must continuously balance tolerance toward commensals and defense against pathogens.

Despite these findings, several limitations should be considered. First, this study used a carcinoma-derived oral epithelial cell line, which may not fully reflect the physiological responses of primary oral epithelial cells. Therefore, validation using primary cells or more physiologically relevant models would strengthen these findings. Second, antimicrobial peptide analysis was based on mRNA expression; thus, translation into protein expression and functional peptide activity was not directly assessed. Future studies should also evaluate whether pLF can inhibit biofilm formation by key oral pathogens such as *Streptococcus mutans* [29]. Furthermore, given the role of TLR-MyD88 signaling in periodontal bone resorption, it would be valuable to investigate whether pLF influences osteoclast differentiation and alveolar bone loss [30,31,32].

Overall, these findings provide mechanistic insight into how pLF modulates epithelial immune responses. The ability of pLF to regulate inflammatory signaling while enhancing antimicrobial-related gene expression suggests its potential role in modulating epithelial innate immune functions in the oral environment. Further studies using more complex in vitro models and in vivo systems are required to confirm these effects and clarify their physiological relevance.

## 5. Conclusions

The lipoteichoic acid fraction derived from *L. plantarum* K8 (pLF) exhibits both anti-inflammatory and antimicrobial effects in human oral epithelial cells through mechanisms involving TLR2-related signaling. pLF treatment induced the expression of IRAK-M, a negative regulator of TLR signaling, which was accompanied by suppression of NF-κB activation and a significant reduction in proinflammatory cytokines, including IL-6, IL-8, and CCL2, indicating attenuation of inflammatory signaling pathways at the epithelial level. In parallel, pLF increased the mRNA expression of antimicrobial peptides such as human β-defensin 1 and β-defensin 2, suggesting enhancement of epithelial innate immune defense mechanisms. Functionally, pLF-treated epithelial cells demonstrated increased antibacterial activity against both Gram-positive and Gram-negative oral pathogens, including *S. aureus* and *P. gingivalis*. Collectively, these results demonstrate the pLF modulates both inflammatory signaling and innate antimicrobial defense responses in human oral epithelial cells through TLR2-involved regulatory pathways, highlighting its biological activity as a postbiotic-derived fraction in regulating epithelial immune functions in the oral environment. 

## Data Availability

The data presented in this study are available on request from the corresponding authors.

## Supplementary Materials

The following supporting information can be downloaded at: https://www.mdpi.com/article/10.3390/microorganisms14061255/s1, Table S1. Primer sequences used for qPCR analysis.

## References

[1] Pihlstrom B.L., Michalowicz B.S., Johnson N.W. (2005). Periodontal diseases. Lancet.

[2] Hajishengallis G. (2015). Periodontitis: From microbial immune subversion to systemic inflammation. Nat. Rev. Immunol..

[3] Kinane D.F., Stathopoulou P.G., Papapanou P.N. (2017). Periodontal diseases. Nat. Rev. Dis. Primers.

[4] Marsh P.D. (2006). Dental plaque as a biofilm and a microbial community. J. Clin. Periodontol..

[5] Scully C. (2008). Aphthous ulceration. N. Engl. J. Med..

[6] Akintoye S.O., Greenberg M.S. (2014). Recurrent aphthous stomatitis. Dent. Clin. N. Am..

[7] Nguyen T., Brody H., Radaic A., Kapila Y. (2021). Probiotics for periodontal health—Current molecular findings. Periodontol. 2000.

[8] Lebeer S., Vanderleyden J., De Keersmaecker S.C.J. (2008). Genes and molecules of lactobacilli supporting probiotic action. Microbiol. Mol. Biol. Rev..

[9] Kim A.R., Ahn K.B., Yun C.H., Park O.J., Perinpanayagam H., Yoo Y.J., Kum K.Y., Han S.H. (2019). *Lactobacillus plantarum* lipoteichoic acid inhibits oral multispecies biofilm. J. Endod..

[10] Ahn K.B., Baik J.E., Park O.J., Yun C.H., Han S.H. (2018). Lipoteichoic acid inhibits *Streptococcus mutans* biofilm formation. PLoS ONE.

[11] Dobson A., Cotter P.D., Ross R.P., Hill C. (2012). Bacteriocin production: A probiotic trait?. Appl. Environ. Microbiol..

[12] Plaza-Díaz J., Ruiz-Ojeda F.J., Gil-Campos M., Gil Á. (2019). Mechanisms of action of probiotics. Adv. Nutr..

[13] Salminen S., Collado M.C., Endo A., Hill C., Lebeer S., Quigley E.M.M., Sanders M.E., Shamir R., Swann J.R., Szajewska H. (2021). The International Scientific Association of Probiotics and Prebiotics (ISAPP) consensus statement on the definition and scope of postbiotics. Nat. Rev. Gastroenterol. Hepatol..

[14] Aguilar-Toalá J.E., Garcia-Varela R., Garcia H.S., Mata-Haro V., Gonzalez-Cordova A.F., Vallejo-Cordoba B., Hernandez-Mendoza A. (2018). Postbiotics: An evolving term within the functional foods field. Trends Food Sci. Technol..

[15] Kim W.-J., Jung G., Kim T., Hurh B.-S., Kim H., Soung D.Y. (2024). Heat-killed *Lactobacillus paracasei* stimulates β-defensins in oral keratinocytes. Microorganisms.

[16] Piqué N., Berlanga M., Miñana-Galbis D. (2019). Health benefits of heat-killed probiotics. Int. J. Mol. Sci..

[17] Takeuchi O., Akira S. (2010). Pattern recognition receptors and inflammation. Cell.

[18] Kawai T., Akira S. (2010). TLR signaling. Nat. Immunol..

[19] Kim H.G., Kim N.R., Gim M.G., Lee J.M., Lee S.Y., Ko M.Y., Kim J.Y., Han S.H., Chung D.K. (2008). Lipoteichoic acid isolated from *Lactobacillus plantarum* inhibits LPS-induced TNF-α production. J. Immunol..

[20] Kobayashi K., Hernandez L.D., Galán J.E., Janeway CAjr-Medzhitov R., Flavell R.A. (2002). IRAK-M is a negative regulator of Toll-like receptor signaling. Cell.

[21] Liew F.Y., Xu D., Brint E.K., O’Neill L.A.J. (2005). Negative regulation of toll-like receptor-mediated immune responses. Nat. Rev. Immunol..

[22] Lawrence T. (2009). The NF-κB pathway in inflammation. Cold Spring Harb. Perspect. Biol..

[23] Campos J., Pires M.F., Sousa M., Camps C., da Cost C.F.F.A., Sampaio-Maia B. (2023). Unveiling the relevance of the oral cavity as a *Staphylococcus aureus* colonization site and potential source of antimicrobial resistance. Pathogens.

[24] Menzies B.E., Kenoyer A. (2006). Staphylococcus aureus infection induces β-defensin expression. Infect. Immun..

[25] Ganz T. (2003). Defensins: Antimicrobial peptides of innate immunity. Nat. Rev. Immunol..

[26] Diamond G., Beckloff N., Weinberg A., Kisich K.O. (2009). The roles of antimicrobial peptides in innate host defense. Curr. Pharm. Des..

[27] Dale B.A., Fredericks L.P. (2005). Antimicrobial peptides in the oral environment: Expression and function. J. Dent. Res..

[28] Gorr S.-U. (2009). Antimicrobial peptides in periodontal innate defense. Periodontol. 2000.

[29] Koo H., Falsetta M.L., Klein M.I. (2013). The exopolysaccharide matrix: A virulence determinant of cariogenic biofilm. J. Dent. Res..

[30] Huang X., Bao J., Yang M., Li Y., Liu Y., Zhai Y. (2024). The role of *Lactobacillus plantarum* in oral health: A review of current studies. J. Oral Microbiol..

[31] Hong J., Son M., Shin J., Kim H., Chung D.K. (2023). Nanoparticles of *Lactiplantibacillus plantarum* K8 reduce *Staphylococcus aureus* respiratory infection and tumor necrosis factor alpha and interferon gamma induced lung inflammation. Nutrients.

[32] Jeong J., Kang B.-H., Ju S., Park N.Y., Kim D., Dinh N.T.B., Lee J., Rhee C.Y., Cho D.H., Kim H. (2024). *Lactiplantibacillus plantarum* K8 lysates regulate hypoxia-induced gene expression. Sci. Rep..

