# Supplementary material for: Lipoteichoic Acid Fraction from Lactiplantibacillus plantarum K8 Attenuates Inflammatory Responses and Promotes Antimicrobial Defense in Oral Epithelial Cells
Source: Microorganisms. 2026 Jun 2;14(6):1255. doi: 10.3390/microorganisms14061255 (PMC13303281; doi:10.3390/microorganisms14061255)
Supplement: Supplementary file 1 [file microorganisms-14-01255-s001.zip › microorganisms-4314469-supplementary.pdf]

Primer	Forward (5’ →3’)	Reverse (5’ →3’)
hTLR1	CTTCTGTTTTTGTGGCCAGGG	GGTGCCCAATATGCCTTTGTTA
hTLR2	TTCCCTGGGCAGTCTTGAAC	GGCTTGAACCAGGAAGACGA
hTLR6	TGCAGACAAGTGTGAGGGAC	CTGAACCTGTGTGCTCCTGT
hCCL2	CTGACCCCAAGCAGAAGTGG	CTTGGGTTGTGGAGTGAGTGT
hIL-6	AAGTCCTGATCCAGTTCCTGC	GGCATTTGTGGTTGGGTCAG
hIL-8	ACACTGCGCCAACACAGAA	TTCTCCACAACCCTCTGCAC
hIRAKM	ACCATGCTCGGTCATCTGTG	ATGTTCTAGGTGGGACCGGA
hSOCS-1	GTGCACGCAGCATTAACTGG	GGAGGGTACCCACATGGTTC
hSOCS-3	GGGGAGTACCACCTGAGTCT	TGTGGTTGCTATCGTCCCAC
hABIN1	GGAGTTCAACCGACTGGCAT	CTTCCGAAGCTGCTCACACA
hA20	CAGTCTGCAGTCTTCGTGGC	GGTGTGATCTCTCTTGGCGG
hCYLD	ATGGTTCTACACAGCCACCC	TTTTCAGCAACGTGGTGTCC
hBD1	CTGCCTGCCCGATCTTTACC	CACTCCCAGCTCACTTGCAG
hBD2	CATCAGCCATGAGGGTCTTGT	ACAGGATCGCCTATACCACCA
hGAPDH	AAGAAGGTGGTGAAGCAGGC	TGGGTGTCGCTGTTGAAGTC

Table S1. Primer sequences used for qPCR analysis
